# CD4^+^CD25^+/high^CD127^low/-^ regulatory T cells are enriched in rheumatoid arthritis and osteoarthritis joints—analysis of frequency and phenotype in synovial membrane, synovial fluid and peripheral blood

**DOI:** 10.1186/ar4545

**Published:** 2014-04-17

**Authors:** Babak Moradi, Philipp Schnatzer, Sébastien Hagmann, Nils Rosshirt, Tobias Gotterbarm, Jan Philippe Kretzer, Marc Thomsen, Hanns-Martin Lorenz, Felix Zeifang, Theresa Tretter

**Affiliations:** 1 University Clinic of Heidelberg, Clinic for Orthopedics and Trauma Surgery, Schlierbacher Landstr. 200a, 69118 Heidelberg, Germany; 2 Klinikum Baden-Baden, Lilienmattstraße 5, 76530 Baden-Baden, Germany; 3 University Clinic of Heidelberg, Department of Medicine V Div. of Rheumatology, Im Neuenheimer Feld 410, 69120 Heidelberg, Germany

## Abstract

**Introduction:**

CD4^+^CD25^+/high^CD127^low/-^ regulatory T cells (Tregs) play a crucial role in maintaining peripheral tolerance. Data about the frequency of Tregs in rheumatoid arthritis (RA) are contradictory and based on the analysis of peripheral blood (PB) and synovial fluid (SF). Because Tregs exert their anti-inflammatory activity in a contact-dependent manner, the analysis of synovial membrane (SM) is crucial. Published reports regarding this matter are lacking, so we investigated the distribution and phenotype of Tregs in concurrent samples of SM, SF and PB of RA patients in comparison to those of osteoarthritis (OA) patients.

**Methods:**

Treg frequency in a total of 40 patients (18 RA and 22 OA) matched for age and sex was assessed by flow cytometry. Functional status was assessed by analysis of cell surface markers representative of activation, memory and regulation.

**Results:**

CD4^+^ T cells infiltrate the SM to higher frequencies in RA joints than in OA joints (*P* = 0.0336). In both groups, Tregs accumulate more within the SF and SM than concurrently in PB (*P* < 0.0001). Relative Treg frequencies were comparable in all compartments of RA and OA, but Treg concentration was significantly higher in the SM of RA patients (*P* = 0.025). Both PB and SM Tregs displayed a memory phenotype (CD45RO^+^RA^-^), but significantly differed in activation status (CD69 and CD62L) and markers associated with Treg function (CD152, CD154, CD274, CD279 and GITR) with only minor differences between RA and OA.

**Conclusions:**

Treg enrichment into the joint compartment is not specific to inflammatory arthritis, as we found that it was similarly enriched in OA. RA pathophysiology might not be due to a Treg deficiency, because Treg concentration in SM was significantly higher in RA. Synovial Tregs represent a distinct phenotype and are activated effector memory cells (CD62L^-^CD69^+^), whereas peripheral Tregs are resting central memory cells (CD62L^+^CD69^-^).

## Introduction

Rheumatoid arthritis (RA) is an autoimmune disease characterized by a chronic relapsing–remitting inflammation primarily of peripheral joints. The tissue-destructive inflammation affects all joint structures, leading eventually to total joint destruction. The pathology of the disease is characterized by disturbed immune regulation with predominance of inflammatory cells on the one hand and defective peripheral immune tolerance on the other [1]. These immunopathologic events occur predominantly within the joints and particularly in the synovial membrane (SM) as the main site of mononuclear cell infiltration. CD4^+^ T cells comprise a large proportion of the inflammatory cells recruited into the RA synovium and contribute significantly to synovial inflammation [2]. In contrast to these inflammatory CD4^+^ T cells, another T-cell subset, known as naturally occurring regulatory T cells (Tregs), has been shown to play an essential role in establishing the balance between pro- and anti-inflammatory mechanisms in the periphery and maintaining self-tolerance, both in rodents and in humans [1,3-5]. The ability of Tregs to suppress T-cell responses, and thereby to regulate immune reactions, ascribes to them a key role in the pathophysiology of autoimmune diseases and makes them an interesting target for treatment [2,6,7]. The suppressive capacity of human CD4^+^ T cells was first shown to be contained within CD4^+^ cells expressing high levels of CD25, the α chain of the IL-2 receptor [8]. Previous research has demonstrated that low expression of the IL-7 receptor α chain (CD127), in combination with CD25, allows the consistent and specific identification of viable CD4^+^CD25^+/high^CD127^low/-^ Tregs and facilitates their discrimination from activated CD4^+^CD25^+^CD127^+^ effector T cells [9-11]. Researchers in a number of studies have analysed the presence of Tregs in RA [12-21]. Apart from contradicting results regarding the analysis of peripheral blood (PB), accumulating evidence indicates that Tregs are enriched in the synovial fluid (SF) of RA joints [12-15,17,18,20]. In contrast, the presence and phenotype of Tregs in the immunological organ of the joint, the SM, remains largely unstudied. This is of special interest because Tregs develop their suppressive capacity in a contact-dependent manner [8,9,14]. Researchers in only two studies have analysed synovial biopsies and suggested a much lower frequency of Tregs in synovial tissue than in SF [18,20]. These immunohistochemical and quantitative PCR analyses provide preliminary insight into Treg distribution, but the techniques utilized do not allow further phenotypic analysis of synovial Tregs and limit comparability to SF and PB quantitative flow cytometry data. Furthermore, the comparison to equivalent samples from non-autoimmune-driven joint disease is missing. These data are essential to understanding RA-specific pathophysiology.

In our present study, we examined the frequency and phenotypes of CD4^+^CD25^+/high^CD127^low/-^ Tregs in the three compartments (PB, SF and SM) of RA patients in order to map the distribution of Tregs between the periphery and the target organ. The data derived from RA patient samples were compared to those from age- and sex-matched samples of patients with OA as a nonautoimmune control group. To the best of our knowledge, this study is the first to show that Treg enrichment into the joint is not specific to inflammatory arthritis. RA pathophysiology is probably not due to a lack of Tregs, because Treg concentration in the SM of RA was significantly higher than in OA. Furthermore, we show that peripheral and synovial Tregs show significant differences regarding activation status and markers associated with Treg function.

## Methods

### Study population

The characteristics of the study population are summarised in Table 1. RA and OA were determined according to the 1987 criteria of the American Rheumatism Association [22]. None of the OA patients had signs of a systemic inflammatory disease based on analysis of CRP and leukocyte counts. All patients were scheduled for knee replacement surgery. The ethics committee of the University of Heidelberg approved this study. Informed consent for participation was obtained from all patients.

**Table 1:** **Characteristics of the study population**^**a**^

**Characteristics**	**RA**	**OA**
Number of patients (M/F)	18 (11/7)	22 (13/9)
Age (yr)	67.5 ± 8	66.7 ± 8.3
Duration of disease (yr)	14 ± 7	15 ± 6
DAS28 score	4.5 ± 1.2	n.a.
RF-positive, *n* (%)	10 (55.6)	n.a.
CRP (mg/L)	17 ± 9	5.1 ± 3
ESR (mm/h)	23 ± 12	4 ± 2
DMARD, *n* (%)	14 (77.8)	0
Anti-TNF treatment, *n* (%)	5 (27.8)	0
Glucocorticoids, *n* (%)	1 (5.6)	0
NSAID, *n* (%)	16 (88.9)	20 (90.1)

^a^CRP, C-reactive protein; DAS28, Disease Activity Score in 28 joints; DMARD, Disease-modifying antirheumatic drug; ESR, Erythrocyte sedimentation rate; n.a., Not applicable; NSAID, Nonsteroidal anti-inflammatory drug; OA, Osteoarthritis; RA, Rheumatoid arthritis; RF, rheumatoid factor; TNF, tumour necrosis factor. Values are mean ± SD unless otherwise indicated.

### Sample collection

SF and SM samples from knee joints were collected during knee surgery. SF was removed prior to arthrotomy by needle aspiration into ethylenediaminetetraacetic acid (EDTA)–containing tubes (Sarstedt, Nümbrecht, Germany). SM was taken from the suprapatellar pouch intraoperatively. PB samples were obtained concurrently and collected in EDTA-containing tubes prior to surgery.

### Cell preparation

SF samples were treated with bovine testicular hyaluronidase (10 mg/ml; Sigma-Aldrich, St Louis, MO, USA) for 30 minutes at 37°C, and cells were washed twice with phosphate-buffered saline (PBS). Single SM samples were freed from other tissue components and rinsed twice with PBS. SM was then minced finely with scissors and digested with collagenase B (1 mg/ml; Roche Applied Science, Indianapolis, IN, USA) and bovine testicular hyaluronidase IV (2 mg/ml) at 37°C for 2 hours in RPMI 1640 culture medium (Invitrogen, Carlsbad, CA, USA) supplemented with 10 μg/ml penicillin/streptomycin (Invitrogen) 10% foetal calf serum (Biochrom AG Biotechnologie, Berlin, Germany). The cell suspension was filtered through a 100-μm filter (BD Biosciences, San Diego, CA, USA) and a 40-μm-pore-size sieve (EMD Millipore, Billerica, MA, USA) to remove any undigested tissue. The filtered cell suspension was washed twice with PBS. Mononuclear cells (PBMCs, SFMCs and SMMCs) were isolated from EDTA anticoagulated whole blood, SF and SM cell suspension samples using Ficoll-Paque™ PLUS density gradient centrifugation (GE Healthcare Life Sciences, Pittsburgh, PA, USA). T cells were isolated from PB, SF and SM mononuclear cells by MACS bead separation (CD3 MicroBeads; Miltenyi Biotec, San Diego, CA, USA) based on previously described protocols [13]. The volume and weight of all samples were measured, and total cell numbers were determined after mononuclear cell separation and CD3 isolation.

### Flow cytometry analysis

Multicolour flow cytometry was used to identify the T-cell subsets and expression of cell surface markers. All monoclonal antibodies (mAbs) were obtained from BD Biosciences unless stated otherwise. In brief, MACS bead separation–isolated T cells were washed twice in staining buffer, blocked with FcR blocking reagent (Miltenyi Biotec) and then stained for 30 minutes at 4°C with fluorescein isothiocyanate (FITC)–labelled mAb against CD4 (clone RPA-T4), phycoerythrin (PE)-labelled mAb against CD25 (clone M-A251) and peridinin chlorophyll protein complex cychrome 5.5 (PerCP-Cy5.5)–labelled mAb against CD127 (clone RDR5; eBioscience, San Diego, CA, USA). For intracellular staining, cells were stained for cell surface markers with CD4-FITC, CD25-allophycocyanin (CD25-APC) (clone 2A3) and CD127-PerCP-Cy5.5, then fixed and permeabilised with the human FoxP3 buffer set (BD Biosciences) according to the manufacturer’s instructions and stained with PE-labelled mAb against FoxP3 (clone 259D/C7). The analysis of activation markers was performed with PE-labelled mAb specific for one of the following cell surface markers: CD45RA (clone HI100; BioLegend, San Diego, CA, USA), CD45R0 (clone UCHL1; BioLegend), CD69 (clone FN50), CD152 (cytotoxic T-lymphocyte antigen 4 (CTLA-4), clone BN13), CD154 (CD40L; clone 89-76), CD274 (programmed cell death receptor 1 ligand (PD-L1), clone 29E.2A3; BioLegend), CD279 (programmed death receptor 1 (PD-1), clone MIH4) and CD62L (L-selectin, clone DREG-56). After being stained for surface molecules, the cells were washed again and analysed with a FACSCalibur flow cytometer (BD Biosciences). A total of 10^5^ events were collected and analysed using the FlowJo software program (TreeStar, Ashland, OR, USA).

### Gating strategy and definition of cell populations

Cells were gated for lymphocytes on the basis of forward and side scatter profiles and further gated for CD4 expression. By using the cell surface markers CD25 and CD127, the Treg population was identified as CD4^+^CD25^+/high^CD127^-/low^ Tregs. The cutoff for all cell surface markers was established based on isotype controls. The CD4^+^ cells with the highest level of CD25 staining were defined as CD4^+^CD25^high^ cells and appeared as a tail to the right from the major CD4^+^ cell population, as previously shown [8]. The CD4^+^CD25^+/high^CD127^-/low^ Treg population was distinct and clearly separable from other cells as previously described [9-11]. For the analysis of activation markers, the CD4^+^CD25^+/high^CD127^-/low^ Treg population was gated as described previously and further analysed for each PE-labelled cell surface marker. Expression of activation marker and mean fluorescence intensity (MFI) was determined.

### Statistical analysis

The demographic parameters of the OA and RA groups were compared using an unpaired *t*-test for parametric data (age and body mass index) and the χ^2^ test for proportions (sex). The unpaired *t*-test was used for analysis of Treg frequency between OA and RA groups, and the paired *t*-test was used for comparisons between concurrent PB, SF and SM samples. Differences in the expression of activation markers were analysed by performing the Mann–Whitney *U* test. Correlation analysis was performed using the Pearson correlation coefficient. All *P*-values reported herein are two-tailed. A *P*-value <0.05 was considered to show a statistically significant difference. Statistical analysis was performed using GraphPad Prism version 5 software (GraphPad Software, La Jolla, CA, USA).

## Results

### Higher accumulation of CD4^+^ T cells in the affected joints of rheumatoid arthritis patients

In order to assess T-cell distribution between the periphery and the joints, we analysed the absolute cell counts of CD4^+^ T cells in concurrent samples of PB, SF and SM by flow cytometry (Table 2). Within each study group, comparison of PB with SF and SM revealed that PB contained significantly higher concentrations of CD4^+^ T cells. Furthermore, the concentration of CD4^+^ T cells was significantly higher in the SM than in the SF. The comparison between RA and OA revealed that the SM of RA patients displayed a significantly higher accumulation of CD4^+^ T cells than age- and sex-matched OA controls (RA: 378 ± 250 cells/μg, OA: 240 ± 140 cells/μg; *P* = 0.0336). No significant differences were observed in SF and PB.

**Table 2:** **Frequency of CD4+ T cells and CD4**^**+**^**CD25**^**+/high**^**CD127**^**low/-**^** regulatory T cells in the peripheral blood and synovial fluid and synovial membrane joint samples in rheumatoid arthritis and osteoarthritis patients**^**a**^

		**RA**	**OA**	***P*-values**						
				**RA:OA**	**RA**	**RA**	**RA**	**OA**	**OA**	**OA**
					**PB:SF**	**PB:SM**	**SF:SM**	**PB:SF**	**PB:SM**	**SF:SM**
PB	Sample size (*n*)	18	22							
	Sample volume (ml)	7 ± 0	7 ± 0							
	Concentration of CD4^+^ T cells (cells /μl)	730 ± 210	690 ± 90	0.424	<0.0001^b^	<0.0001^b^		<0.0001^b^	<0.0001^b^	
		(610 to 900)	(540 to770)							
	CD4^+^CD25^high^ (%total CD4^+^ T cells)	4.88 ± 2.04	4.4 ± 1.59	0.417	0.0072^b^	0.0381^b^		<0.0001^b^	0.004^b^	
		(2 to 9.4)	(1.9 to 8)							
	CD4^+^CD25^+/high^CD127^low/-^ (% total CD4^+^ T cells)	6.7 ± 1.8	7.6 ± 1.8	0.135	0.0006^b^	0.0128^b^		0.0001^b^	0.0091^b^	
		(4 to 11)	(5 to 11)							
	CD4^+^CD25^+/high^CD127^low/-^ (cells/μl)	48.9 ± 7.8	52.4 ± 4	0.099	<0.0001^b^	0.0004^b^		<0.0001^b^	<0.0001^b^	
		(30 to 99)	(29 to 85)							
SF	Sample size (*n*)	17	22							
	Sample volume (ml)	15.41 ± 12.65	11.21 ± 6.81							
		(4.3 to 38)	(2.2 to 23.6)							
	Concentration of CD4^+^ T cells (cells/μl)	70 ± 170	40 ± 90	0.479	<0.0001^b^		0.0001^b^	<0.0001^b^		<0.0001^b^
		(30 to 540)	(10 to 480)							
	CD4^+^CD25^high^ (% total CD4^+^ T cells)	9.97 ± 6.27	9.63 ± 4.12	0.7371	0.0072^b^		0.2449	<0.0001^b^		0.1086
		(3.4 to 19)	(3.3 to 22.6)							
	CD4^+^CD25^+/high^CD127^low/-^ (% total CD4^+^ T cells)	11.8 ± 5.5	12.4 ± 5	0.727	0.0006^b^		0.1226	0.0001^b^		0.4549
		(5 to 22)	(4 to 26)							
	CD4^+^CD25^+/high^CD127^low/-^ (cells/μl)	8.3 ± 9.4	5 ± 4.5	0.153	<0.0001^b^		<0.0001^b^	<0.0001^b^		<0.0001^b^
		(8to90)	(4 to 100)							
SM	Sample size (*n*)	18	22							
	Sample volume (mg)	3.45 ± 1.18	3.72 ± 1.3							
		(2.2 to 5.9)	(1.95 to 5.73)							
	Concentration of CD4^+^ T cells (cells/μg)	378 ± 250	240 ± 140	0.0336^b^		<0.0001^b^	0.0001^b^		<0.0001^b^	<0.0001^b^
		(10 to 610)	(10 to 340)							
	CD4^+^CD25^high^ (% total CD4^+^ T cells)	7.21 ± 3.9	7.5 ± 4.8	0.8354		0.0381^b^	0.2449		0.004^b^	0.1086
		(2 to 18.4)	(2.5 to 26)							
	CD4^+^CD25^+/high^CD127^low/-^ (% total CD4^+^ T cells)	9.4 ± 3.8	11.2 ± 5.9	0.266		0.0128^b^	0.1226		0.0091^b^	0.4549
		(5 to 17)	(2.4 to 26)							
	CD4^+^CD25^+/high^CD127^low/-^ (cells/μg)	35.5 ± 12	27 ± 11	0.025^b^		0.0004^b^	<0.0001^b^		<0.0001^b^	<0.0001^b^
		(24 to 89)	(14 to 73)							

^a^OA, Osteoarthritis; PB, Peripheral blood; RA, Rheumatoid arthritis; SF, Synovial fluid; SM, Synovial membrane. Collected sample volumes and calculated concentrations of CD4^+^ T cells are shown. Frequencies of CD4^+^CD25^high^ and of CD4^+^CD25^+/high^CD127^low/-^ cells are shown as the percentage of CD4^+^ T cells and for CD4^+^CD25^+/high^CD127^low/-^ cells also as cell concentration (total cell counts per microlitre or per microgram) for PB, SF and SM in RA and OA patients. Values shown are mean ± SD (range) unless otherwise stated. ^b^Statistically significant differences.

### Equal frequency of CD4^+^CD25^+/high^CD127^-/low^ Tregs in peripheral blood of RA and OA patients

**Figure 1:**
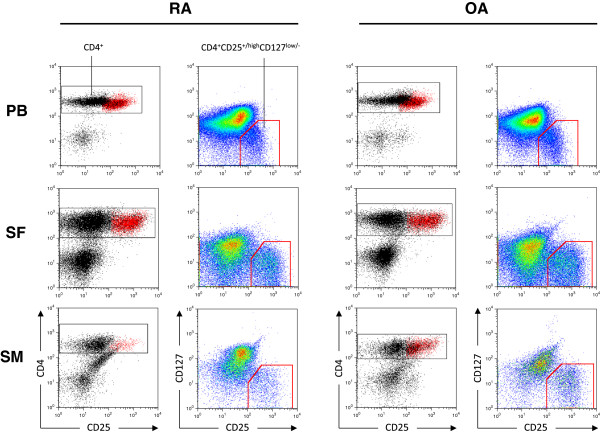
**Flow cytometry analysis of CD4**^**+**^**CD25**^**+/high**^**CD127**^**low/- **^**regulatory T cells in all compartments from rheumatoid arthritis and osteoarthritis patients.** Peripheral blood (PB), synovial fluid (SF) and synovial membrane (SM) CD4^+^ T cells were analysed by flow cytometry after staining with fluorescein isothiocyanate (FITC)–labelled monoclonal antibody (mAb) against CD4 (clone RPA-T4), phycoerythrin-labelled mAb against CD25 (clone M-A251) and peridinin chlorophyll protein complex cychrome 5.5–labelled mAb against CD127 (clone RDR5; eBioscience). Representative dot plots for one RA patient and one OA patient are shown. The cells in these analyses were gated for lymphocytes via their forward and side scatter properties and further for CD4^+^ cells by positive anti-CD4-FITC staining (first column for each group). The red gates in the second columns indicate the CD4^+^CD25^+/high^CD127^low/-^ regulatory T cells. These cells are back-gated and shown as the red CD4^+^CD25^+/high^ population in the first columns of each patient group.

**Figure 2:**
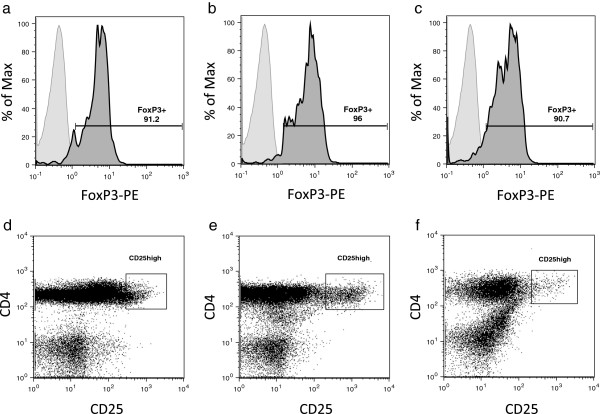
**FoxP3 expression of CD4**^**+**^**CD25**^**+/high**^**CD127**^**low/- **^**T cells in different compartments and gating strategy for CD25**^**high **^**cells.** Samples were stained with anti-CD4–fluorescein isothiocyanate (FITC) (clone 2A3), anti-CD25–allophycocyanin (APC) (clone RDR5) and anti-CD127–peridinin chlorophyll protein complex cychrome 5.5 (anti-CD127-PerCP) (clone RDR5) to identify CD4^+^CD25^+/high^CD127^low/-^cells. After undergoing fixation and permeabilisation, cells were further stained with phycoerythrin-labelled FoxP3 (clone 259D/C7). The cells in these analyses were gated for CD4^+^CD25^+/high^CD127^low/-^regulatory T cells, as shown in Figure 1. Cells stained with isotype controls are shadowed in the histograms. The boldface line represents the FoxP3^+^ T cells. Four samples were analysed for each compartment. Representative histograms for **(a)** peripheral blood (PB), **(b)** synovial fluid (SF) and **(c)** synovial membrane (SM) are shown. The numbers in the histograms indicate the percentage of gated cells expressing FoxP3^+^. **(d)** to **(f)** Staining pattern for CD4-FITC and CD25-APC is shown. Representative dot plots of one patient are shown. Gated areas indicate the CD25^high^ cell populations in **(d)** PB, **(e)** SF and **(f)** SM.

We evaluated the frequency of CD4^+^CD25^+/high^CD127^-/low^ Tregs by flow cytometry (Figure 1). Intracellular FoxP3 staining of the CD4^+^CD25^+/high^CD127^-/low^ cells was performed in four patients, which revealed that the gated cells were highly positive for FoxP3, as shown in Figures 2d to 2f (PB: 91 ± 4%, SF: 94 ± 8%, SM: 90 ± 7%). We analysed the relative frequency of Tregs and plotted these data as the percentage of CD4^+^ T cells and the absolute Treg count, which are given as the concentration for each compartment in Table 2. The relative and absolute frequencies of CD4^+^CD25^+/high^CD127^-/low^ Tregs in the peripheral blood of RA and OA patients were comparable, without any significant differences. In order to allow comparability to previous studies, we also determined the frequency of CD4^+^CD25^high^ cells as shown in Figures 2d to 2f. The same pattern was shown when gated for CD4^+^CD25^high^ cells (Table 2).

### CD4^+^CD25^+/high^CD127^-/low^ Tregs are enriched in joints of RA and OA patients

We further analysed SF and SM samples of the same patients in order to map the distribution of Tregs between periphery and the affected joints. CD4^+^CD25^+/high^CD127^-/low^ Tregs were significantly enriched in the SF and SM joint samples of both patient groups in comparison to PB (Table 2), with SF displaying the highest percentage of CD4^+^CD25^+/high^CD127^low/-^ Tregs, followed by SM and then PB. No significant differences between RA and OA Treg percentages were evident for the three compartments. Gating for CD4^+^CD25^high^ cells led to smaller Treg numbers in all compartments, but the distribution and statistical significance between compartments and the two study groups remained the same. Regarding total Treg numbers, the highest Treg concentration in both groups was present in PB, followed by SM and then SF, which is due to the higher CD4^+^ T-cell count in these compartments. The comparison between the two groups showed a significantly higher Treg concentration in the SM of RA patients (*P* = 0.025).

### Correlation analysis of Treg frequencies between compartments and with clinical data

In order to map Treg compartmentalization and analyse the relationship between the PB and the joint compartment, we performed correlation analysis of Treg frequency. Only small correlations were detectable between PB and SF or SM (*r* = 0.145 to 0.154, respectively), and a medium correlation was found between SF to SM (*r* = 0.263 to 0.424), but these correlations were not significant (*P* = 0.059 to 0.361, respectively). We found no significant correlation between Treg frequency and activation markers and clinical parameters, such as sociodemographic and laboratory parameters, disease activity and medication.

### Phenotype analysis of CD4^+^CD25^+/high^CD127^low/-^ Tregs in RA and OA patients

**Figure 3:**
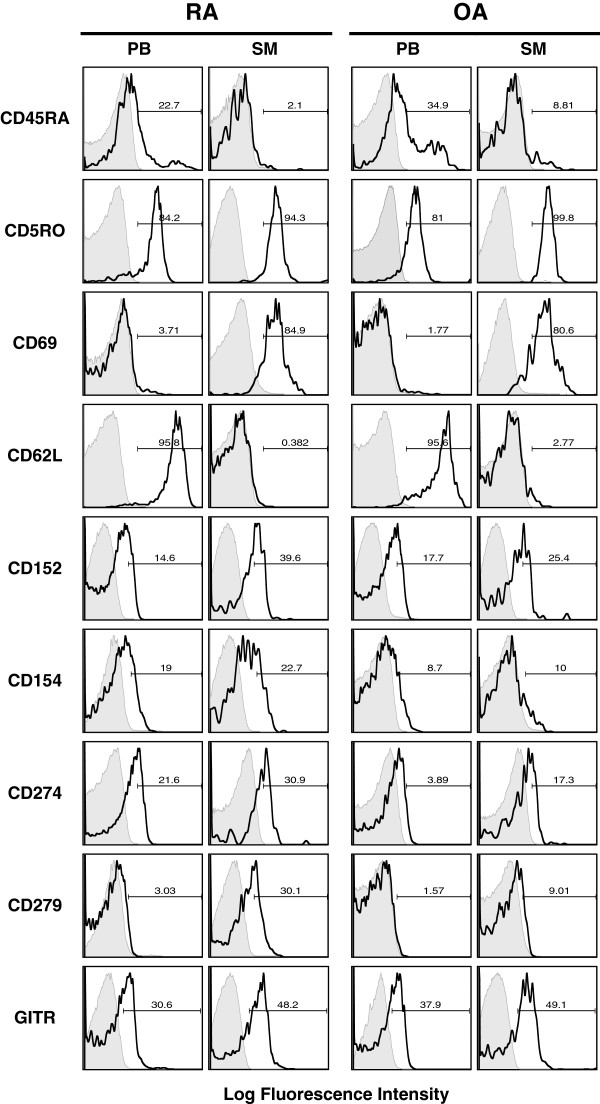
**Phenotype characteristics of CD4**^**+**^**CD25**^**+/high**^**CD127**^**low/- **^**T cells in rheumatoid arthritis and osteoarthritis patients in different compartments.** Samples were stained with anti-CD4–fluorescein isothiocyanate (clone 2A3), anti-CD25–allophycocyanin (clone RDR5), and anti-CD127–peridinin chlorophyll protein complex cychrome 5.5 (clone RDR5) to identify CD4^+^CD25^+/high^CD127^low/-^ cells. The cells were further stained for the following surface markers: CD45RA (clone HI100; BioLegend), CD45R0 (clone UCHL1; BioLegend), CD69 (clone FN50), CD152 (cytotoxic T-lymphocyte antigen 4 (CTLA-4), clone BN13), CD154 (CD40L, clone 89-76), CD274 (PD-L1, clone 29E.2A3; BioLegend), CD279 (PD-1, clone MIH4) and CD62L (L-selectin, clone DREG-56). Cells stained with isotype controls are shadowed in the histograms. The boldface line represents the CD4^+^CD25^+/high^CD127^low/-^ T cells. GITR, Glucocorticoid-induced tumour necrosis factor receptor; OA, Osteoarthritis; PB, Peripheral blood; RA, Rheumatoid arthritis; SM, Synovial membrane.

A panel of cell surface markers was selected to characterise the phenotype and activation status of joint-derived Tregs (SF and SM) in comparison to corresponding cells from PB in both patient groups (Table 3, Additional file 1: Table S1, and Figure 3). SF- and SM-derived Tregs displayed the same phenotype as that described in Table 3, which is why the comparison described next focuses on the analysis of PB and SM samples. The majority of the PB CD4^+^CD25^+/high^CD127^low/-^ Tregs were memory cells. PB Tregs expressed CD45RO in 83% of RA patients and 81.4% OA patients. CD45RO is associated with proliferative responses to recall antigens. The opposite expression profile was evident for CD45RA, which is a marker for naive T cells. In this study, 22.9% of the PB CD4^+^CD25^+/high^CD127^low/-^ Tregs of RA patients expressed CD45RA, compared to 34.9% in OA patients. This pattern was also evident for synovial Tregs, with an even higher expression of CD45RO and a significantly lower expression of CD45RA. Only a small proportion of PB Tregs in RA and OA expressed CD69, a common marker for early T-cell activation. In synovial Tregs, CD69 expression significantly increased to about 80%, confirming higher T-cell activation, with only small differences observed between the two patient groups. The significantly higher activation status of synovium-derived Tregs was further supported by the expression profile of L-selectin (CD62L). CD62L was expressed by about 90% of PB Tregs in both patient groups and decreased significantly in the SM samples to 2% in RA patients and 3.1% in OA patients (*P* = 0.0293 and *P* = 0.0001, respectively). This finding was also confirmed by MFI (Additional file 1: Table S1). The expression of CD152 (CTLA-4), CD154 (CD40L), CD274 (PD-L1), CD279 (PD-1) and glucocorticoid-induced tumour necrosis factor receptor (GITR) were also evaluated in order to gain insight into the regulatory function of Tregs. CD152, which displayed only low expression levels in PB, increased significantly in SM, with a slightly higher expression in RA patients than in OA patients. The expression level of CD154 in PB Tregs was low in both patient groups and increased slightly in the SM, without any significant difference between RA and OA patients. We observed a significant increase for SM in OA patients when analysed by MFI. In PB, CD274 showed a significantly higher expression level in RA patients (23.5%) than in OA patients (9.8 %) (*P* = 0.0329). In both groups, CD274 expression was increased in synovial Tregs, diminishing the difference seen in PB. The expression profiles of CD279 and GITR showed a similar pattern, with increased expression in SM samples. For CD279, this increase was found to be significant upon comparing PB and SM of RA patients (*P* = 0.0231). Furthermore, synovial Tregs showed a significantly higher expression of CD279 in RA patients than in OA patients (*P* = 0.0239), which was also present when analysed for MFI (*P* = 0.0421). No significant difference in GITR could be detected between the patient groups.

**Table 3:** **Phenotype characteristics of CD4**^**+**^**CD25**^**+/high**^**CD127**^**low/-**^** T-cells from peripheral blood, synovial fluid and synovial membrane of rheumatoid arthritis and osteoarthritis patients**^**a**^

	**RA**			**OA**			***P*-values**			
**Surface marker**	**PB**	**SF**	**SM**	**PB**	**SF**	**SM**	**RA**	**OA**	**PB**	**SM**
							**PB:SM**	**PB:SM**	**RA:OA**	**RA:OA**
CD45RA	22.9 ± 5	5 ± 3	2.8 ± 1	34.9 ± 13.6	6 ± 3	5.6 ± 2.5	0.0059^b^	0.0006^c^	0.0631	0.2377
	21 (19.5 to 30.2)	4 (3 to 7)	2.8 (2 to 3.5)	30.8 (16.8 to 57.4)	7 (2 to 9)	6 (2.1 to 8.8)				
CD45RO	83 ± 14.7	93 ± 7	94.7 ± 5.2	81.4 ± 8.5	92 ± 8	91.9 ± 7.2	0.4	0.0232^b^	0.9554	0.6488
	81 (70 to 99)	92 (87 to 99)	94.9 (89.4 to 99)	82 (60 to 95)	91 (81 to 98)	94.3 (80.9 to 99.8)				
CD69	3.1 ± 1.9	88 ± 2	84.8 ± 0.2	1.9 ± 0.8	82.3 ± 5.3	81.2 ± 4.8	< 0.0001^c^	0.0029^d^	0.1623	0.4746
	2.1 (1.9 to 5.2)	85 (80 to 92)	84.6 (78 to 89)	1.8 (0.9 to 3.7)	84 (77.3 to 88)	82.6 (74.6 to 84.9)				
CD62L	94.6 ± 1.8	5 ± 2	2 ± 1	94.7 ± 4.4	3 ± 1	3.1 ± 0.5	< 0.0001^c^	0.0293^b^	0.8881	0.2480
	94.5 (93.3 to 95.8)	3 (3 to 9)	2 (1.1 to 3.2)	95.3 (80.5 to 99.7)	3.5 (2 to 5)	3.1 (2.8 to 3.5)				
CD152	12.6 ± 14	29.2 ± 12	36.2 ± 3.3	13 ± 6.5	31.5 ± 11	32.5 ± 8.2	0.0477^b^	0.0049^d^	0.7552	0.8530
	5.7 (3.2 to 28.8)	33 (33 to 40)	36 (33 to 39)	16.3 (1 to 19)	30 (24 to 41)	32.5 (25 to 39.6)				
CD154	13.5 ± 6.3	11 ± 5.7	15.1 ± 8.8	8.8 ± 5.4	12 ± 8	13.4 ± 8.2	0.5892	0.2863	0.2361	0.8486
	13.4 (7 to 19.5)	13 (6 to 18)	15.1 (7 to 23)	8.5 (1.4 to 19)	11 (5 to 20)	10 (7.5 to 18)				
CD274	23.5 ± 2.8	25 ± 7	27.6 ± 5.7	9.8 ± 10.4	25 ± 7	24.1 ± 7.9	0.3758	0.0313^b^	0.0329^b^	0.5541
	23 (21 to 26)	32 (20 to 29)	31 (21 to 31)	6 (0.5 to 40)	26 (17 to 34)	24.1 (17 to 30.9)				
CD279	3.3 ± 3.3	23.2 ± 12	22.6 ± 11	5.4 ± 6.5	9 ± 9	7.9 ± 10	0.0231^b^	0.6677	0.8127	0.0239^b^
	3 (0.2 to 6.7)	24 (14 to 32)	22.6 (15 to 30.1)	2.2 (0 to 23.1)	8 (2 to 15)	8 (0.8 to 15)				
GITR	30.3 ± 22.7	42.5 ± 7.3	45.5 ± 6.8	40.5 ± 12.8	49.2 ± 12	47.4 ± 11.9	0.329	0.173	0.636	0.8114
	31.4 (7 to 52.4)	43 (39 to 51)	45.1 (38.9 to 52)	43.8 (18.4 to 59.1)	13.9 (33 to 59)	11.94 (30 to 58.7)				

^a^GITR, Glucocorticoid-induced tumour necrosis factor receptor–related protein; OA, Osteoarthritis; PB, Peripheral blood; RA, Rheumatoid arthritis; SF, Synovial fluid; SM, Synovial membrane. Significant differences: ^b^*P* < 0.05; ^c^*P* < 0.001; ^d^*P* < 0.01. CD4^+^CD25^+/high^CD127^low/-^ regulatory T cells from PB, SF and SM were stained for cell surface markers as indicated in the left column. The percentage of cells positive for each surface marker is presented as mean ± SD and median and range. Data are representative of three RA patients) and five OA patients. Statistical analysis is shown for PB and SM because SF values did not differ those for SM.

## Discussion

Data about the frequency of Tregs in PB of RA vary throughout the literature, in which it is described as decreased [13,14,16,17,21], similar [12,15] and increased [14,19] compared to healthy controls. This controversy is partly due to the lack of one specific Treg marker and the resulting differences in the identification of Tregs (CD25^+^ vs. CD25^high^). The transcription factor FoxP3, which is required for Treg development, has improved the specificity of Treg detection, but its intracellular location does not allow separation of viable cells [23]. The establishment of CD127 as an additional surface marker, in combination with CD25, has been found to facilitate a consistent quantitative identification of viable CD4^+^CD25^+/high^CD127^low/-^ Tregs, which are highly positive for FoxP3 [9-11]. Only one previous study has provided quantitative data regarding CD4^+^CD25^+/high^CD127^low/-^ Tregs in the PB of RA patients. The investigators showed lower frequency in active RA and similar Treg frequency in RA patients in remission compared to controls [21]. In our present study, the mean frequency of CD4^+^CD25^+/high^CD127^low/-^ Tregs in PB samples was comparable between RA and OA patients. It seems that the analysis of circulating Tregs has not described the disease-specific parameters consistently enough to draw conclusions. This raises the question whether the analysis of Treg frequency at the site of inflammation might provide a clearer pattern of RA pathology. Researchers in a few studies have analysed the SF of affected RA joints and showed an increase in Treg frequency compared to PB samples taken concurrently from those patients and healthy controls [12-15,17,20,24]. This accumulation into the affected RA joints ran counter the hypothesis of a reduced Treg presence in RA pathophysiology. But due to the lack of SF control samples in these studies, it remained unresolved if the increase of Treg frequencies in RA SF might turn out as a reduced frequency when compared to non-RA SF controls. The inclusion of SF from OA patients in our study shows for the first time that Treg enrichment into the joints is not a feature specific to RA, but is also present in the joints of nonautoimmune OA patients. Furthermore, our data disprove the hypothesis of a lack of Tregs in RA because we show that Treg frequency in SF is comparable between RA and OA patients. The fact that Tregs develop their suppressive activity in a contact-dependent manner, as well as that soluble factors alone are unable to exert suppression, highlights the relevance of further investigation of the SM [8,9,14]. Only two studies have analysed the SM for the presence of FoxP3 by immunohistochemistry and qPCR [18,20]. Those two studies have suggested a much lower frequency of Tregs in SM than in SF and PB, favouring the hypothesis that, in RA patients, the SM lacks sufficient Treg numbers and thus these patients would benefit therapeutically from an increase in Tregs. In order to further evaluate this hypothesis, we analysed concurrent samples of SM from RA and OA patients and provide for the first time flow cytometry–derived quantitative data. Our results are contradictory to the common assumption that Treg frequency is lower in SM than in PB. Both groups in our study exhibited significant enrichment of Tregs in the SF and SM compared to concurrent PB. When analysed for absolute Treg numbers, the SM showed a significantly higher Treg concentration than SF in OA and RA patients. Additionally, the comparison to OA samples revealed that Treg concentration is significantly higher in the SM of RA patients. Our results indicate that Treg deficiency is not the underlying mechanism of RA development. Instead, the significantly higher CD4^+^ T-cell infiltration of RA joints suggests that Tregs are either counterbalanced by effector T cells in the joint or functionally impaired. Herrath *et al*. recently showed that the inflammatory milieu in RA joints reduces the ability of Tregs to cope with the overwhelming number of inflammatory cells [24]. This favours the hypothesis that the suppressive activity of Tregs can be altered and counterbalanced by activated responder T cells in the joint, which are less susceptible to suppression as their counterparts from PB [14].

The discrepancy between our results and those reported in the previous two studies might be due to factors such as study population, sample collection and utilization of different techniques. Apart from the older age of our study population, the demographics and clinical parameters of our study are comparable to those of these previous studies [18,20]. We received all samples at the time of joint surgery, which differs from the study of Raghavan *et al*., who collected SF samples mainly during RA flares [20]. How this can affect Treg frequency and status remains unknown. In studies comprising longitudinal samples taken from patients with psoriatic arthritis, spondyloarthropathy and juvenile idiopathic arthritis, the frequency of Tregs was found to be relatively stable between flares and remissions [10,12,13]. The two-dimensional character of immunohistochemistry and the analysis of tissue slices provides information about a rather limited cell number. The distribution of infiltrating cell populations appears not to be homogeneous throughout the joint. Regions with high inflammatory infiltrates might lead to an underestimation of Tregs due to an overrepresentation of total CD4^+^ T cells. In our present study, flow cytometry was the method of choice because it allows for determination of mean frequency of distinct cell populations and the simultaneous analysis of the expression of multiple surface markers in individual cells. Even qPCR does not allow examination of individual cells and a combination of markers.

One underlying hypothesis about Treg enrichment into the affected joints of RA and other inflammatory diseases is that Tregs are either attracted or generated because of ongoing inflammation [25]. Because our results demonstrate that Treg enrichment is also evident in OA joints, this hypothesis needs reevaluation. It could be argued that OA pathology is also accompanied by low-level chronic synovial inflammation [26]. Taking this into account, the accumulated Treg presence in the joints of both patient groups can be understood as an attempt of the immune system to control the inflammatory responses. The faster and much more severe progression of joint destruction in RA could be due to the complex inflammatory environment that might lead to impaired Treg function.

Another hypothesis is that compartmentalization rather than inflammation is the driving force. The physiology of the joint compartment differs from the periphery, and Treg compartmentalization has been shown to be tissue- and organ-specific [17,27]. The low correlation between PB, SF and SM Treg frequency in our study suggests that distinct mechanisms contribute to selective retention and trafficking of Tregs and that the SF and SM cannot be expected to mirror the PB. We hypothesize that the joint compartment contains a higher polarized CD4^+^ T-cell population and, as such, also a higher Treg population compared to PB, where the majority of T cells are naïve and not yet polarized into subsets. Assuming this is true, future studies need to include the simultaneous analysis of inflammatory T-cell subsets in order to map the compartmentalization of effector and regulatory T cells and analyse their balance in the periphery and the joint.

The fact that the joint compartment differs significantly from the periphery became even more evident when the phenotypic analysis of Tregs was performed. In accord with the results of previous studies, our data confirm that peripheral Tregs display a CD45RO^+^CD45RA^-^ memory phenotype [12,28]. This was further increased in SM Tregs, where almost all Tregs presented a memory phenotype. Significant differences between the periphery and the joint were also observed when analysed for activation markers CD69 and CD62L. SM Tregs were CD62L^-^CD69^+^ activated effector memory cells compared to CD62L^+^CD69^-^ resting central memory Tregs in PB. Significant differences were also seen for markers associated with Treg function. CD152 is thought to be essential for the suppressive ability of Tregs [29,30]. Peripheral Tregs constitutively express low surface levels of CD152, with increased and long-lasting expression after stimulation [6,7,14,15,30-33]. In accord with these findings, our data display low expression of surface CD152 in PB, which was significantly increased in the SM, confirming the activated state of synovium-derived Tregs [14]. Murine and human studies have attributed a relevant role for GITR in Treg function, where antibody binding abrogates suppression [34,35]. Our data are in accord with those of previous studies in which researchers have shown low expression of GITR in the periphery [14] and a significant increase in synovial Tregs [6,7,31-34]. Since the intensity of GITR expression has been correlated with the suppressive capacity of the Treg cells [33], the higher expression on synovium-derived Tregs could indicate a higher suppressive capacity of synovial Tregs. The programmed death receptor 1 (PD-1) CD279 is involved in the mechanism of cell-cycle arrest [5,30,36,37]. Its ligand CD274 (PD-L1) was recently shown to deliver a negative signal through the PD-1 receptor, which downregulates T-cell proliferation and cytokine production [38,39]. When analysed in PB, the expression level of CD279 or CD274 did not show any significant difference between RA and OA patients, with low expression levels for both. CD274 showed a significant increase in SM Tregs in both groups, whereas CD279 increased only in the SM of RA patients. Further analyses are necessary to evaluate how these expression levels translate into functional differences between Tregs from different compartments and between RA and OA patients.

## Conclusions

Our data show that CD4^+^ T cells are significantly enriched in the SM of RA patients when compared to OA. CD4^+^CD25^+/high^CD127^low/-^ Tregs accumulation in the affected joints is not disease specific for RA but also evident in OA. Thus, the comparison and normalization of the quantitative data to OA levels is necessary in order to understand RA disease specific pathology, which is the strength of this study. Since relative Treg frequencies did not differ significantly between RA and OA and the Treg concentration in RA SM samples was even significantly higher than in OA, we hypothesize that Treg deficiency is not the underlying mechanism in RA. The significant higher CD4^+^ T cell infiltration of RA joints rather suggests that Tregs are either functionally impaired or counterbalanced by effector T cells in the joint, which may become Treg resistant. Phenotype analysis revealed significant differences between PB and SM Tregs showing that synovial Tregs are activated memory cells compared to resting memory cells in the PB. This underlines the importance of analysing samples from the affected joints and most importantly the SM as the target organ, when studying RA pathophysiology. We think that the simultaneous assessment of synovial effector T-cells and Tregs and their functional properties are pivotal for the understanding or RA pathophysiology. Our results demonstrate for the first time that flow cytometric identification of viable synovial Tregs according to surface marker expression is possible and this opens new avenues for their use in functional studies.

## Abbreviations

CRP: C-reactive protein; DAS28: Disease Activity Score in 28 joints; DMARD: Disease-modifying antirheumatic drug; ESR: Erythrocyte sedimentation rate; GITR: Glucocorticoid-induced tumour necrosis factor receptor; MFI: Mean fluorescence intensity; NSAID: Nonsteroidal anti-inflammatory drug; OA: Osteoarthritis; PB: Peripheral blood; PD-1: Programmed death receptor 1; RA: Rheumatoid arthritis; RF: Rheumatoid factor; SF: Synovial fluid; SM: Synovial membrane; TNF-α: Tumour necrosis factor α; Treg: Regulatory T cell.

## Competing interests

The University of Heidelberg funded this study. The funding source did not have any involvement in the study design, data collection, analysis and interpretation of data, writing of the manuscript or the decision to submit the manuscript for publication. No benefits in any form have been or will be received from any commercial party related directly or indirectly to the subject of this manuscript. The authors declare that they have no competing interests.

## Authors’ contribution

BM and TT were responsible for study conception and design, data acquisition, data analysis and interpretation and manuscript writing. PS was responsible for data acquisition, data analysis and interpretation and critical revision of the manuscript. SH was responsible for data acquisition, data analysis and manuscript writing. NR and TG were responsible for data acquisition and critical revision of the manuscript. JPK and NT were responsible for data analysis and interpretation and critical revision of the manuscript. HML was responsible for study conception and design, data analysis and interpretation and manuscript writing. FZ was responsible for study conception and design, data analysis and interpretation and manuscript writing. All authors read and approved the final manuscript.

## Supplementary Material

Additional file 1: Table S1 Mean fluorescence intensity of activation markers of CD4^+^CD25^+/high^CD127^low/-^ T cells from peripheral blood, synovial fluid and synovial membrane of rheumatoid arthritis and osteoarthritis patients. CD4^+^CD25^+/high^CD127^low/-^ regulatory T-cells from peripheral blood (PB), synovial fluid (SF) and synovial membrane (SM) were stained for cell surface markers as indicated in the left column. Mean fluorescence intensity for each surface marker is presented as mean ± SD or median and range. Data are representative of three rheumatoid arthritis (RA) to five osteoarthritis (OA) patients. Statistical analysis are shown for PB and SM because SF values did not differ from SM values. Significant differences are indicated by asterisks: *P < 0.05; **P < 0.01. Click here for file
