# Supplementary material for: CD4+CD25+/highCD127low/- regulatory T cells are enriched in rheumatoid arthritis and osteoarthritis joints—analysis of frequency and phenotype in synovial membrane, synovial fluid and peripheral blood
Source: Arthritis Res Ther. 2014 Apr 17;16(2):R97. doi: 10.1186/ar4545 (PMC4060198; doi:10.1186/ar4545)
Supplement: Additional file 1: Table S1 — Mean fluorescence intensity of activation markers of CD4+CD25+/highCD127low/- T cells from peripheral blood, synovial fluid and synovial membrane of rheumatoid arthritis and osteoarthritis patients. CD4+CD25+/highCD127low/- regulatory T-cells from peripheral blood (PB), synovial fluid (SF) and synovial membrane (SM) were stained for cell surface markers as indicated in the left column. Mean fluorescence intensity for each surface marker is presented as mean ± SD or median and range. Data are representative of three rheumatoid arthritis (RA) to five osteoarthritis (OA) patients. Statistical analysis are shown for PB and SM because SF values did not differ from SM values. Significant differences are indicated by asterisks: *P < 0.05; **P < 0.01. [file ar4545-S1.docx]

**Supplemental Table 1: Mean fluorescence intensity of activation markers of CD4^+^CD25^+/high^CD127^low/-^** **T cells from PB, SF and SM of RA and OA patients.**

|  | **RA** | | | **OA** | | | ***p*-values** | | | |
| --- | --- | --- | --- | --- | --- | --- | --- | --- | --- | --- |
| **Surface marker** | **PB** | **SF** | **SM** | **PB** | **SF** | **SM** | **RA**  **PB:SM** | **OA**  **PB:SM** | **PB**  **RA:OA** | **SM**  **RA:OA** |
| **CD45RA** | 19052 ± 19810  10577  (4370 – 52336) | 2600 ± 340  2903  (2230 – 4100) | 2262 ± 151  2262  (2155– 2369) | 5809 ± 3563  5349  (2241 – 18287) | 2850 ± 740  2600  (1853 – 3900) | 2625 ± 890  2510  (1672 – 3809) | 0.3088 | **0.0094**** | **0.0287*** | 0.6386 |
| **CD45RO** | 3466 ± 1178  3308  (2375 – 4715) | 3100 ± 650  3320  (2790 – 4150) | 3338 ± 596  3553  (2663 – 3797) | 4057 ± 1074  3793  (2679 – 6717) | 3250 ± 700  3207  (2461 – 3890) | 3119 ± 655  3108  (2464 – 3797) | 0.999 | 0.119 | 0.4674 | 0.7163 |
| **CD69** | 2244 ± 833.7  1961  (1588 – 3182) | 3150 ± 236  3540  (3200 – 3590) | 3333 ± 213.5  3333  (3182 – 3484) | 2215 ± 349.5  2168  (1692– 2859) | 3590 ± 350  3610  (3380 – 3800) | 3485 ± 231  3507  (3182 – 3743) | 0.1830 | **0.0029**** | 0.6954 | 0.6386 |
| **CD62L** | 19430 ± 5679  19430  (14512 – 24348) | 1790 ± 110  1695  (1450 – 1901) | 1516 ± 85  1516  (1456 – 1765) | 19151 ± 6870  18734  (8228 – 37140) | 1679 ± 150  1670  (1575 – 1800) | 1671 ± 133  1670  (1576 – 1765) | **0.0136*** | **0.0294*** | 0.994 | 0.3015 |
| **CD152** | 692 ± 69  722  (613 – 741) | 833 ± 22  810  (797 – 860) | 805 ± 8  805  (797 – 813) | 715 ± 106  685  (591 – 1000) | 824 ± 11  815  (797 – 820) | 807 ± 7.7  809  (797 – 813) | **0.0481*** | 0.0651 | 0.995 | 0.8516 |
| **CD154** | 928.3 ± 88.56  887  (868 – 1030) | 1010 ± 60  1020  (986 – 1120) | 1040 ± 52  1032  (986 – 1110) | 909 ± 52.1  917  (782– 981) | 1030 ± 76  1025  (994 – 1100) | 1040 ± 63  1023  (980– 1100) | 0.2286 | **0.0085**** | 0.9553 | 0.989 |
| **CD274** | 1675 ± 460  1430  (1389 – 2206) | 1590 ± 150  1580  (1510 – 1785) | 1572 ± 116  1572  (1457 – 1688) | 1418 ± 157  1408  (1241 – 1743) | 1522 ± 102  1525  (1450 – 1680) | 1572 ± 94  1572  (1457 – 1688) | 0.7 | 0.08 | 0.374 | 0.8516 |
| **CD279** | 636.7 ± 78.3  593  (590 – 727) | 1080 ± 280  1090  (780 – 1450) | 1118 ± 283  1127  (782 – 1435) | 641 ± 89.3  608  (567 – 861) | 575 ± 190  579  (350 – 783) | 581 ± 185  599  (345 – 782) | 0.0571 | 0.6326 | 0.7526 | **0.0421*** |
| **GITR** | 629.3 ± 110  624  (522 – 742) | 600 ± 180  570  (458 – 789) | 601 ± 170  564  (453 – 787) | 703 ± 199  641  (466 – 1090) | 711 ± 81  709  (635 – 780) | 706 ± 79  703  (629 – 787) | 0.8226 | 0.5386 | 0.6956 | 0.3872 |
